# Differences in Susceptibility to Heat Stress along the Chicken Intestine and the Protective Effects of Galacto-Oligosaccharides

**DOI:** 10.1371/journal.pone.0138975

**Published:** 2015-09-24

**Authors:** Soheil Varasteh, Saskia Braber, Peyman Akbari, Johan Garssen, Johanna Fink-Gremmels

**Affiliations:** 1 Division of Veterinary Pharmacy, Pharmacology and Toxicology, Utrecht University, Utrecht, The Netherlands; 2 Division of Pharmacology, Utrecht Institute for Pharmaceutical Sciences, Faculty of Science, Utrecht University, Utrecht, The Netherlands; 3 Nutricia Research, Utrecht, The Netherlands; Bose Institute, INDIA

## Abstract

High ambient temperatures negatively affect the human well-being as well as animal welfare and production. The gastrointestinal tract is predominantly responsive to heat stress. The currently available information about the multifaceted response to heat stress within different parts of the intestine is limited, especially in avian species. Hence, this study aims to evaluate the heat stress-induced sequence of events in the intestines of chickens. Furthermore, the gut health-promoting effect of dietary galacto-oligosaccharides (GOS) was investigated in these heat stress-exposed chickens. Chickens were fed a control diet or diet supplemented with 1% or 2.5% GOS (6 days) prior to and during a temperature challenge for 5 days (38–39°C, 8h per day). The parameters measured in different parts of the intestines included the genes (qPCR) HSF1, HSF3, HSP70, HSP90, E-cadherin, claudin-1, claudin-5, ZO-1, occludin, TLR-2, TLR-4, IL-6, IL-8, HO-1, HIF-1α) and their associated proteins HSP70, HSP90 and pan-cadherin (western blots). In addition, IL-6 and IL-8 plasma concentrations were measured by ELISA. In the jejunum, HSF3, HSP70, HSP90, E-cadherin, claudin-5, ZO-1, TLR-4, IL-6 and IL-8 mRNA expression and HSP70 protein expression were increased after heat stress exposure and a more pronounced increase in gene expression was observed in ileum after heat stress exposure, and in addition HSF1, claudin-1 and HIF-1α mRNA levels were upregulated. Furthermore, the IL-8 plasma levels were decreased in chickens exposed to heat stress. Interestingly, the heat stress-related effects in the jejunum were prevented in chickens fed a GOS diet, while dietary GOS did not alter these effects in ileum. In conclusion, our results demonstrate the differences in susceptibility to heat stress along the intestine, where the most obvious modification in gene expression is observed in ileum, while dietary GOS only prevent the heat stress-related changes in jejunum.

## Introduction

Heat stress is one of the most relevant environmental stressors in poultry production worldwide [1]. It has been suggested that in modern poultry genotypes the rapid growth rate is responsible for the reduction in heat tolerance due to the higher metabolic activity [2–4]. In turn, today’s chickens seem to be particularly susceptible to high environmental temperatures and suffer from multiple patho-physiological alterations, such as immune dysregulation, gut barrier dysfunction and cellular oxidative stress after heat exposure, resulting in decreased productivity and increased susceptibility to infectious diseases and higher mortality [5–7]. Response to environmental stressors, including heat stress, starts with the phosphorylation and trimerisation of heat shock factors (HSF) and these trimers translocate to the nucleus and bind the so-called heat shock elements in the promoter region of heat shock protein (HSP) genes, mediating HSP gene transcription. HSPs play a pivotal role in repair and protection of the internal environment by assisting protein refolding and by promoting the degradation of misfolded proteins [8,9]. A general symptom of heat stress is the disturbance of the balance between the production of reactive oxygen species and the cellular antioxidant defenses, resulting in oxidative stress [4,10]. The gastrointestinal tract is primarily responsive to heat stress and a variety of changes can be observed, including alterations in the microbiota and an impairment of intestinal barrier integrity [10,11]. These changes allow the translocation of luminal antigens and pathogens through the intestinal epithelium and facilitate the response of the innate immune system by exaggerating the extent of Toll-like receptor (TLR) signaling, ultimately leading to the development of intestinal inflammation and damage [12,13]. In addition, HSPs are recognized by TLRs in many cell types and can directly initiate an inflammatory response [14–16]. Moreover, the intestinal barrier integrity can be affected by different cytokines [17] and an increase in pro-inflammatory cytokines, like IL-6 and IL-8, has been observed in intestinal epithelial cells after barrier disruption [18,19]. It is also known that the up-regulation of HSPs, and in particular HSP70, is considered to be a protective mechanism as they can also inhibit the expression of pro-inflammatory cytokines [20,21]. The heat stress-induced damages within the intestine is a complex process and needs to be investigated in order to identify intervention strategies and hence, this study focused on the assessment of typical alterations in the expression of a number of genes and their corresponding proteins, such as HSFs, HSPs, adherens junctions (AJ) and tight junctions (TJ), TLRs, cytokines/chemokines and oxidative stress markers, which are all related to the hypothetical cascade of events occurring in different parts of the intestine from broilers upon heat stress exposure. Previous intervention strategies to alleviate heat stress in poultry mainly focused on improvement in antioxidant capacity attributed to supplementation with selenium, vitamins and different unsaturated acids, including α-lipoic acid [22–25]. In contrast, limited information is available about promoting gut health and intestinal barrier integrity in heat stress susceptible chickens. Food supplementation with prebiotics, including galacto-oligosaccharides (GOS) are known to support the maintenance of the gut homeostasis, not only by increasing the beneficial bacteria population, but also by directly improving gut barrier functions and gut-associated immunity [19,26–28]. Therefore, this study included also experiments aiming to evaluate the possible protective role of GOS against the heat stress-induced alterations in the intestines of chickens.

## Materials and Methods

### Animals and experimental design

Sixty 15-day-old Ross broilers were randomly divided into 6 groups (control group, control group + 1% GOS, control group + 2.5% GOS, heat stress (HS) group, HS group + 1% GOS, HS group + 2.5% GOS). The chickens were housed in two environmentally controlled chicken rooms (control and heat stress room) equipped with 3 special bird units and equipped with a lighting program of 16 h light and 8 h dark per day. After an acclimatization period of 6 days (22–26°C), the control groups maintained the ambient temperature of 22–23°C, while the room temperature for the heat stress group was 38–39°C for 8h during the daylight period for 5 consecutive days, and 22–23°C during the remaining period. All chickens were provided with free access to water and feed. The whole experimental period (day 1–11), the broilers were fed either a standard broiler diet (S1 Table) or the standard diet supplemented with 1% or 2.5% GOS (Research Diet Services, Wijk-bij-Duurstede, The Netherlands). GOS was obtained from FrieslandCampina Domo (Vivinal® GOS syrup, Borculo, The Netherlands) containing oligosaccharides with a degree of polymerisation (dp) of 2–8 with approximately 59% (w/w) galacto-oligosaccharides, 21% (w/w) lactose, 19% (w/w) glucose and 1% (w/w) galactose on dry matter (dry matter of 75%).

The experimental protocol was established in line with the prerequisites of the use of animals in research (DIRECTIVE 2010/63/EU) and had been approved prior to the onset of the experimental trials by the Animal Welfare Committee of the University of Veterinary Medicine Hannover (competent ethics committee of the university) on the uses of animals in research according to BGBl. I S. 1105/2.

### Sample collection

At the end of the experimental period, the animals were sacrificed by cervical dislocation and immediately after decapitation blood was collected. Samples from the duodenum (3 cm after gizzard), jejunum (5 cm before Meckel’s diverticulum), ileum (5 cm before ileo-cecal transition), cecum (proximal part) and colon (5 cm after ileo-cecal transition) were dissected, directly rinsed in phosphate buffer saline (PBS), snap frozen in liquid nitrogen and stored at -80°C for qRT-PCR or western blot analysis. Plasma was derived from blood (~10 ml), harvested by centrifugation (15 min at 1500 × g) and stored at –20°C.

### Quantitative RT-PCR (qRT-PCR) analysis

Intestinal specimens suspended in RNA lysis buffer containing β-mercaptoethanol were homogenized using the TissueLyser (Qiagen, Hilden, Germany) for 1 minute/25 Hz and total RNA was isolated using spin columns based on manufacturer's instructions (Promega, Madison, WI, USA). RNA was reverse-transcribed to cDNA using iScriptTM cDNA Synthesis kit (Bio-Rad Laboratories Inc., Hercules, CA, USA). The PCR reaction mixture, containing iQSYBR Green Supermix (Bio-Rad Laboratories Inc.) was prepared based on manufacturer’s instructions and qRT-PCR analysis was performed using the MyiQ single-colour real time PCR detection system (Bio-Rad Laboratories Inc.) with MyiQ System Software Version 1.0.410 (Bio-Rad Laboratories Inc.). Commercially manufactured gene specific primers (Eurogentec, Seraing, Belgium) were used after confirmation of specificity and efficiency tests by qRT-PCR with dilution series of pooled cDNA at a temperature gradient (55°C to 65°C) for primer-annealing and subsequent melting curve analysis (S2 Table). The mRNA quantity was calculated relative to the expression of β-Actin (ACTB) reference gene.

### Western blot analysis

Approximately, 50 mg of jejunum and ileum specimens (five randomly selected samples per group) were lysed with 500 μl RIPA lysis buffer (Thermo scientific, Rockford, IL, USA) containing protease inhibitors (Roche Applied Science, Penzberg, Germany). Total protein concentration was assessed by a BCA protein assay kit (Thermo scientific) and equal protein amounts of boiled samples were separated by electrophoresis (Criterion^TM^ Gel, 4–20% Tris-HCL, Bio-Rad Laboratories Inc.) and electro-transferred onto polyvinylidene difluoride membranes (Bio-Rad, Veenendaal, The Netherlands). Membranes were blocked with PBS supplemented with 0.05% Tween-20 (PBST) and 5% milk proteins and incubated overnight at 4°C with antibodies for HSP70 (1:2000 Abcam, Cambridge, UK), HSP90 (1:1000, Enzo Life Sciences, Farmingdale, NY, USA), and pan-cadherin (1:1000, Abcam). Membranes were subsequently probed with an anti-β-Actin antibody (1:2000, Cell Signaling, Danvers, MA, USA) to evaluate equality of loading. After washing in PBST, the membranes were incubated with appropriate horseradish peroxidase-conjugated secondary antibodies (1:5000, Dako, Glostrup, Denmark) for 2 h at room temperature. Finally, blots were washed in PBST, incubated with ECL Prime western blotting Detection Reagent (Amersham Biosciences, Roosendaal, The Netherlands) and digital images were obtained with the ChemiDoc^TM^ MP imager (Bio-Rad Laboratories Inc.). Signal intensities were quantified using the ImageJ 1.47 software and the protein expression was normalized with β-Actin and expressed as mean fold change in relation to the control group.

### Cytokine measurement

IL-8 and IL-6 concentrations were measured in plasma and jejunal and ileal homogenates using the CXCL8-ELISA kit (BD Biosciences, Cat.# 555244, San Diego, CA, USA) and IL-6 ELISA kit (MyBiosources, Cat.# MBS037319, San Diego, CA, USA) according to manufacturer’s instructions.

### Statistical analysis

Experimental results are expressed as mean ± SEM of n = 6–10 animals/experiment (qRT-PCR and ELISA) or n = 5 animals/experiment (western blot analysis). Differences between multiple groups (qRT-PCR and ELISA results) were statistically determined by using two-way ANOVA, with Bonferroni posthoc test, while data of the western blot analysis were statistically analyzed by using one-way ANOVA followed by a Bonferroni post-hoc test. Analyses were performed by using GraphPad Prism (version 6.05) (GraphPad, La Jolla, CA, USA) and results were considered statistically significant when p < 0.05. Different lowercase letters on the bars indicate significant differences between groups.

## Results

### The effect of heat stress on HSF1, HSF3, HSP70 and HSP90 mRNA levels is more pronounced in ileum compared to jejunum, while dietary GOS only prevent the heat stress response in jejunum

**Fig 1:**
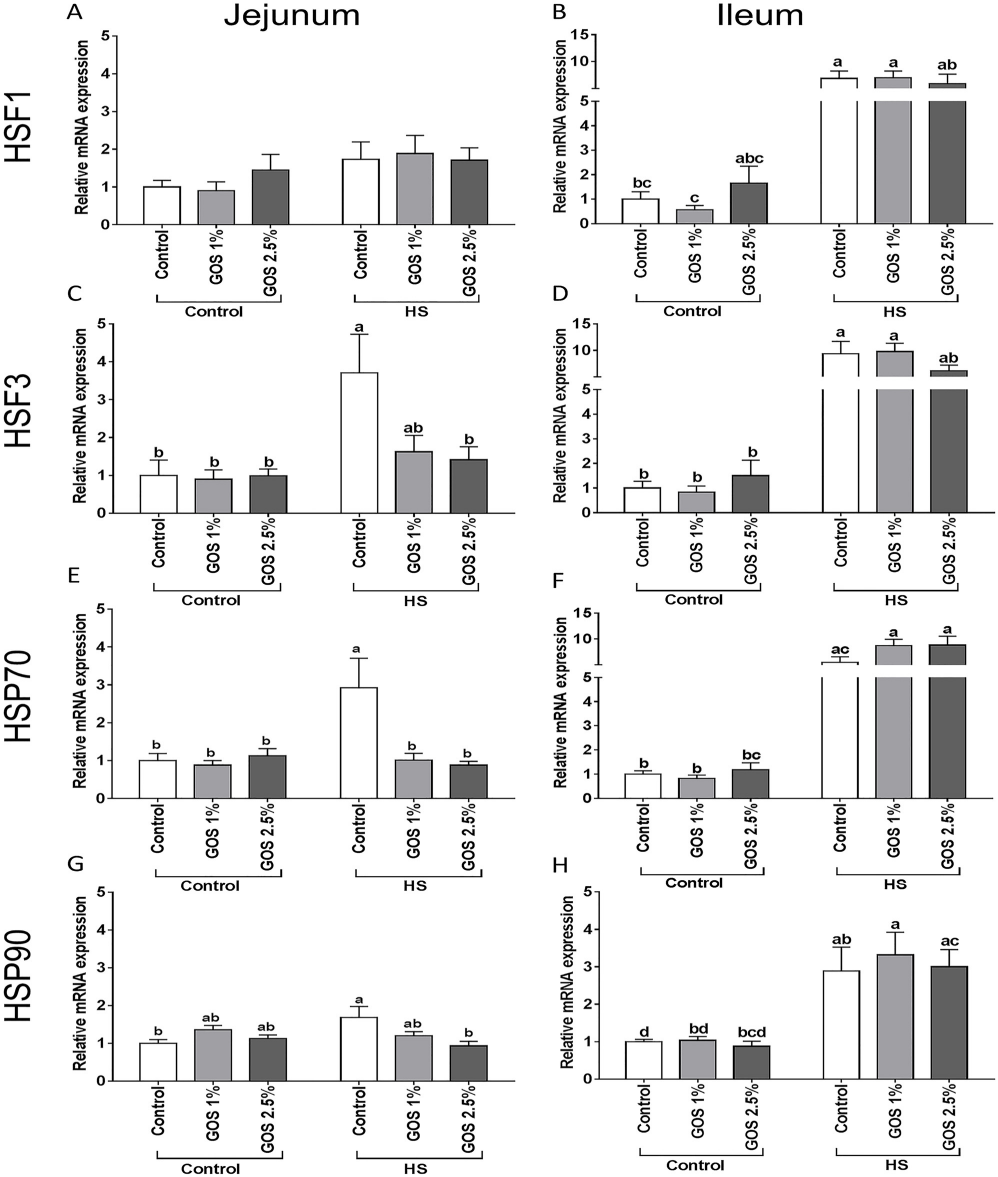
The effect of heat stress exposure on the mRNA expression of HSFs and HSPs in jejunum and ileum of chickens fed a control or GOS diet. Chickens fed a control or GOS (1 or 2.5%) diet for 6 days before being exposed to either control or heat stress conditions for 5 days. mRNA expression levels of HSF1 (A, B), HSF3 (C, D), HSP70 (E, F) and HSP90 (G, H) were evaluated in jejunum (A, C, E, G) and in ileum (B, D, F, H) by qRT-PCR. Results are expressed as relative mRNA expression (fold of control, normalized to β-actin) as mean ± SEM, n = 6–10 animals/experimental group. Different lower-case letters denote significant differences among groups.

Heat stress resulted in a significant mRNA up-regulation of HSF3 in the chicken jejunum (Fig 1C), while in ileum both HSF1 and HSF3 mRNA levels were increased after heat exposure (Fig 1B and 1D). The mRNA expression of HSP70 and HSP90 was significantly up-regulated in both jejunum and ileum of chickens exposed to heat stress compared to control chickens (Fig 1E, 1F, 1G and 1H), although the effect on HSP70 was more pronounced (Fig 1E and 1F). In general, the induction of the heat stress response observed by expression of HSFs and HSPs, was more obvious in the chicken ileum in comparison with the jejunum (Fig 1). In addition, no significant effects on HSP70 and HSP90 mRNA levels were observed in duodenum and colon of chickens exposed to heat stress (S1 Fig). Dietary GOS prevented the heat stress-induced up-regulation of HSF3 (Fig 1C), HSP70 (Fig 1E) and HSP90 (Fig 1G) in the chicken jejunum, while no preventive effect of GOS was observed on the heat stress response in the ileum of chickens exposed to heat stress (Fig 1B, 1D, 1F and 1H).

### HSP70 protein levels are increased in jejunum and ileum after heat stress exposure and dietary GOS prevent this effect in jejunum

**Fig 2:**
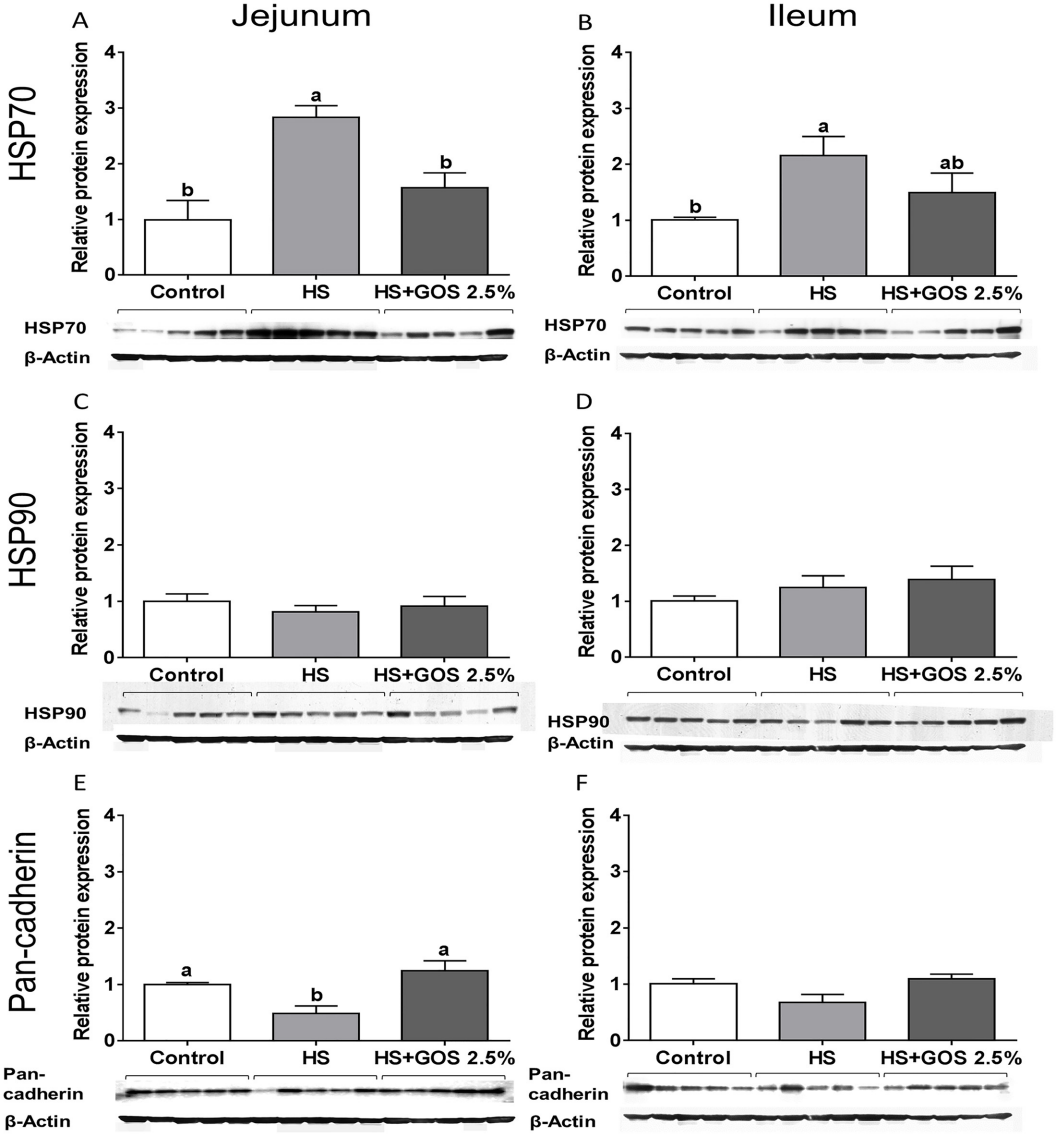
The effect of heat stress exposure on the protein expression of HSPs and pan-cadherin in jejunum and ileum of chickens fed a control or GOS diet. Chickens fed a control or GOS 2.5% diet for 6 days before being exposed to control or heat stress conditions for 5 days. Protein expression levels of HSP70 (A, B), HSP90 (C, D) and pan-cadherin (E, F) were measured in jejunum (A, C, E) and ileum (B, D, F) by western blot analysis (lane 1–5: control chickens, lane 6–10: heat-exposed chickens, lane 11–15: heat-exposed chickens fed a GOS diet). Results are expressed as relative protein expression (fold of control, normalized to β-actin) as mean ± SEM. n = 5 animals/experimental group. Different lower-case letters denote significant differences among groups.

Results indicated that the protein expression of HSP70 was significantly increased in jejunum and ileum of chickens exposed to heat stress (Fig 2A and 2B), whereas the HSP90 protein levels did not change in response to heat stress (Fig 2C and 2D). Dietary GOS could prevent the heat-induced increase in HSP70 protein expression in chicken jejunum (Fig 2A), while in the ileum a small but not significant decrease was observed in the heat-induced HSP70 protein levels (Fig 2B).

### Heat stress modulates the E-cadherin and tight junction protein mRNA expression in the small intestine and dietary GOS prevent this effect in jejunum

**Fig 3:**
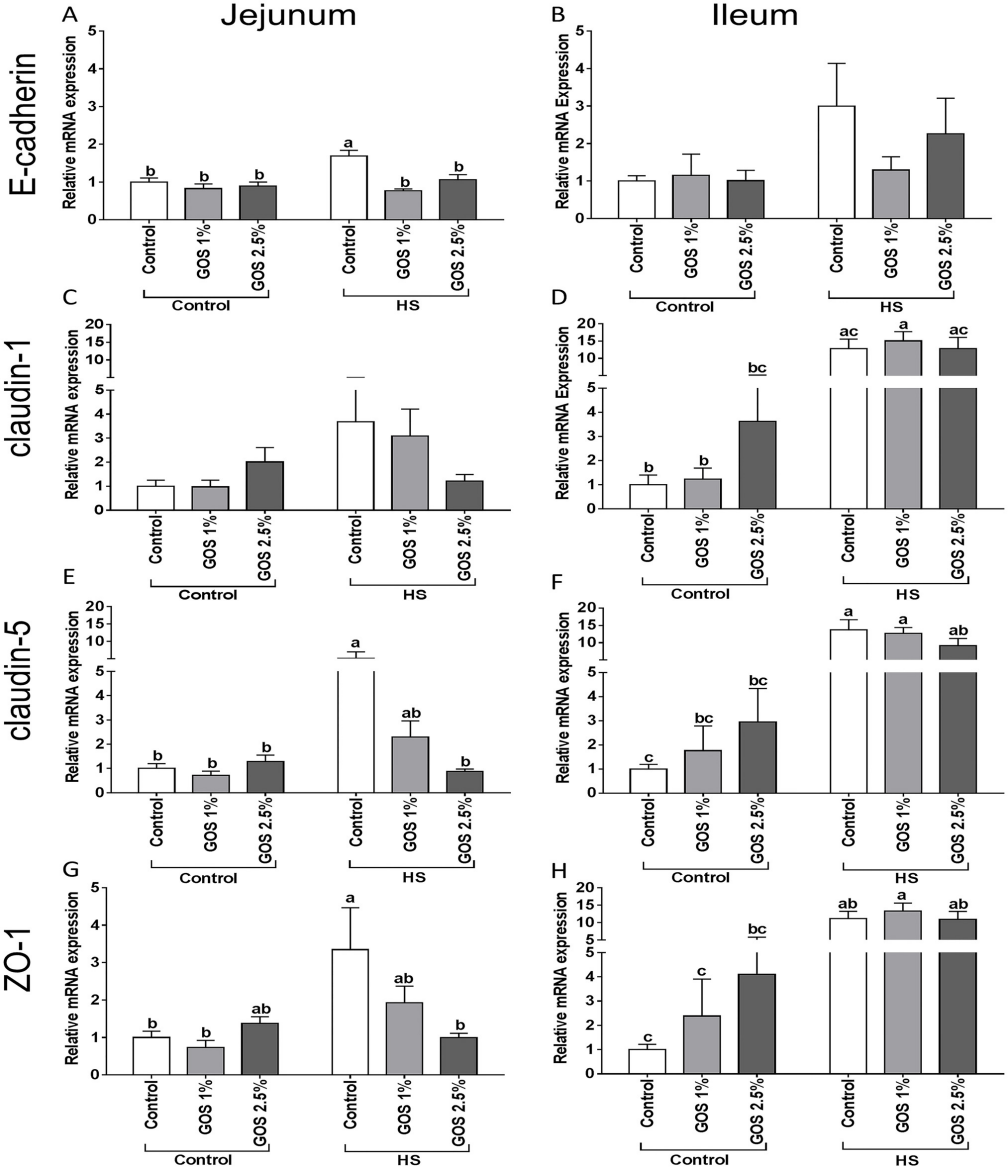
The effect of heat stress exposure on the mRNA expression of AJ and TJ in jejunum and ileum of chickens fed a control or GOS diet. Chickens fed a control or GOS (1 or 2.5%) diet for 6 days before being exposed to either control or heat stress conditions for 5 days. mRNA expression of E-cadherin (A, B), claudin-1 (C, D), claudin-5 (E, F) and ZO-1 (G, H) in jejunum (A, C, E, G) and ileum (B, D, F, H) were evaluated by qRT-PCR. Results are expressed as relative mRNA expression (fold of control, normalized to β-actin) as mean ± SEM, n = 6–10 animals/experimental group. Different lower-case letters denote significant differences among groups.

Heat stress significantly up-regulated the E-cadherin mRNA expression in chicken jejunum (Fig 3A), while in ileum the heat-induced increase in E-cadherin mRNA levels was not statistically significant (p = 0.94) (Fig 3B). The western blot analysis revealed that heat stress decreased the pan-cadherin protein expression in jejunum (Fig 2E), although again no significant effect in the ileum was observed (Fig 2F). The heat-induced effects on both the E-cadherin mRNA expression and pan-cadherin protein expression in jejunum were prevented in chickens fed a GOS diet compared to chickens fed a control diet (Figs 2E and 3A).

Besides E-cadherin, the mRNA expression levels of the TJ proteins claudin-5 (Fig 3E) and zona occludens protein-1 (ZO-1) (Fig 3G) were significantly increased in jejunum of chickens exposed to heat stress. A more pronounced increase in claudin-5 (Fig 3F) and ZO-1 (Fig 3H), but also claudin-1 (Fig 3D) was observed in the ileum after heat stress exposure. Heat stress did not induce any changes in claudin-1 mRNA expression in jejunum (Fig 3C) and occludin mRNA expression in both jejunum and ileum (S2 Fig).

In contrast to the ileum, the heat-induced increase in TJ mRNA expression (claudin-5 and ZO-1) was dose-dependently alleviated in the jejunum of chickens fed a GOS diet (Fig 3E and 3G).

### TLR-4 mRNA levels are increased in jejunum and ileum after heat stress exposure and dietary GOS counteract this effect in jejunum

**Fig 4:**
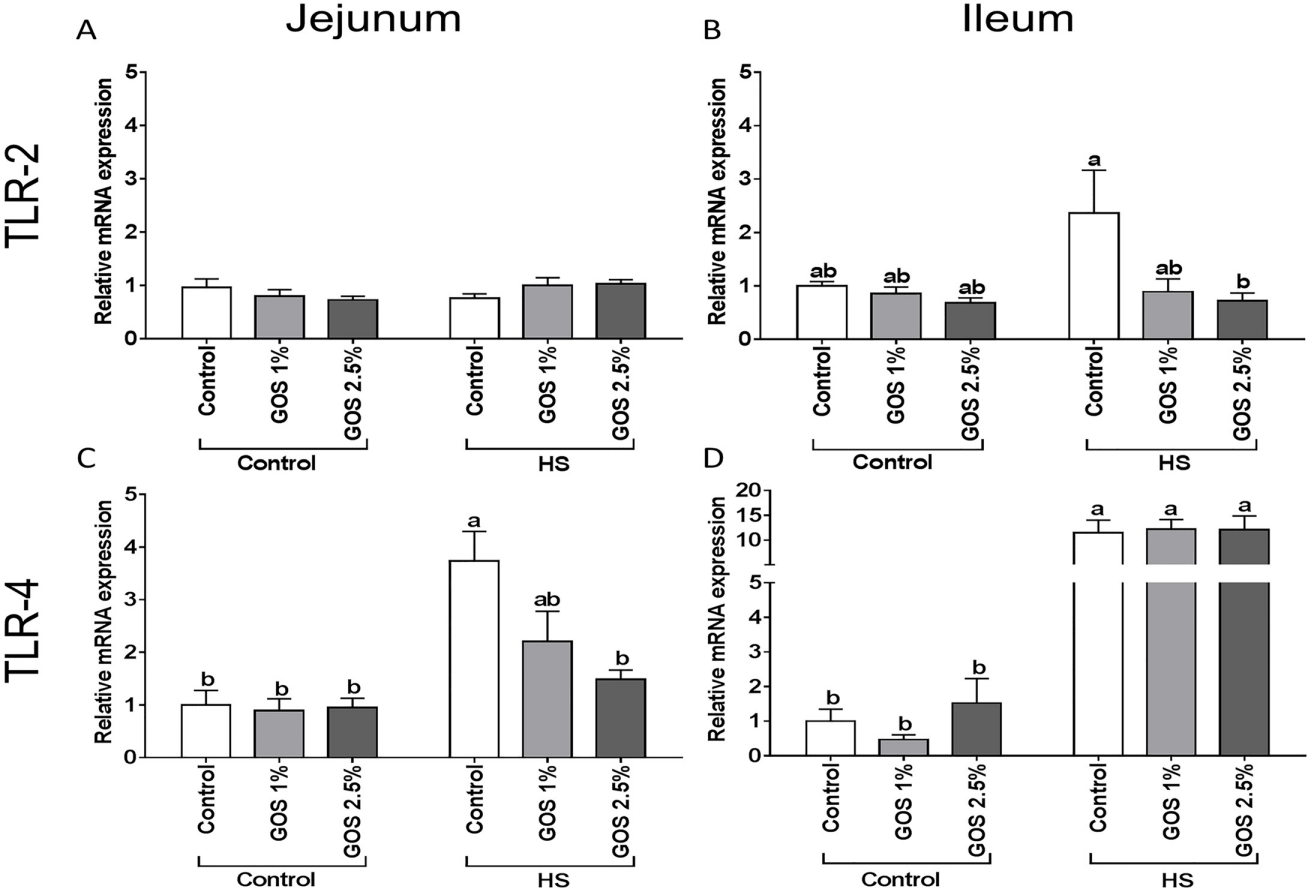
The effect of heat stress exposure on the mRNA expression of TLR-2 and TLR-4 in jejunum and ileum of chickens fed a control or GOS diet. Chickens fed a control or GOS (1 or 2.5%) diet for 6 days before being exposed to either control or heat stress conditions for 5 days. mRNA expression of TLR-2 (A, B) and TLR-4 (C, D) in jejunum (A, C) and ileum (B, D) were evaluated by qRT-PCR. Results are presented as relative mRNA expression (fold of control, normalized to β-actin) as mean ± SEM, n = 6–10 animals/experimental group. Different lower-case letters denote significant differences among groups.

Although no significant differences were detected in TLR-2 mRNA levels between control and heat-exposed chickens in jejunum and ileum (Fig 4A and 4B), the mRNA expression of TLR-4 increased significantly in both jejunum (Fig 4C) and ileum (Fig 4D) after heat exposure. Supplementation of GOS (2.5%) to the diet significantly prevented the heat-induced TLR-4 induction in jejunum (Fig 4C), while GOS did not modulate this effect in the chicken ileum (Fig 4D).

### The up-regulation of IL-6 and IL-8 mRNA expression in jejunum and ileum by heat stress is mitigated by GOS in jejunum

**Fig 5:**
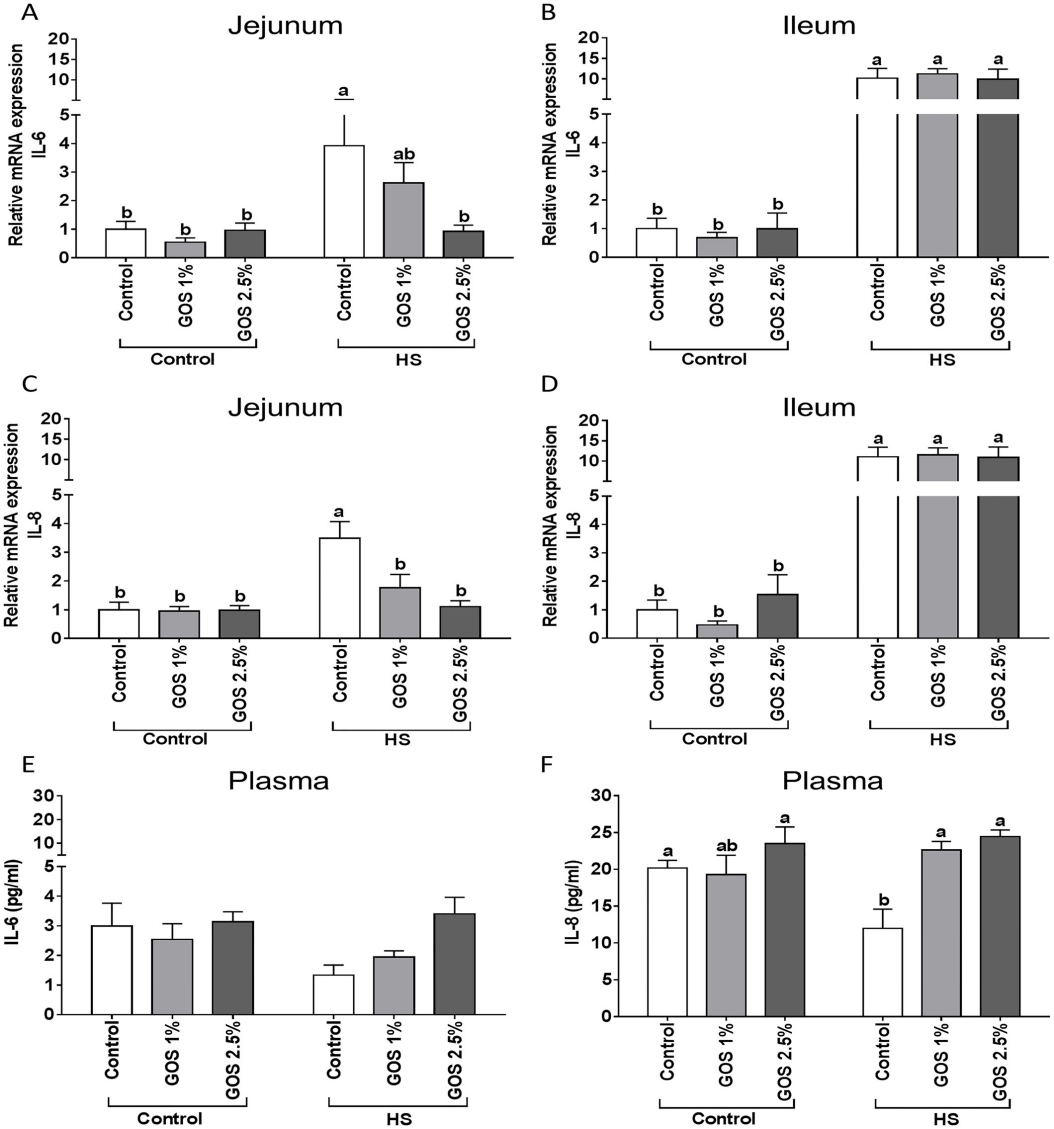
The effect of heat stress exposure on the IL-6 and IL-8 plasma levels and mRNA expression in jejunum and ileum of chickens fed a control or GOS diet. Chickens fed a control or GOS (1 or 2.5%) diet for 6 days before being exposed to either control or heat stress conditions for 5 days. mRNA expression of IL-6 (A, B) and IL-8 (C, D) in jejunum (A, C) and ileum (B, D) evaluated by qRT-PCR. Results are expressed as relative mRNA expression (fold of control, normalized to β-actin) as mean ± SEM. IL-6 (E) and IL-8 (F) secretion in plasma determined by ELISA. Results are expressed in pg/ml as mean ± SEM. n = 6–10 animals/experimental group. Different lowercase letters denote significant differences among groups.

The mRNA expression of the inflammatory markers, IL-6 (Fig 5A and 5B) and IL-8 (Fig 5C and 5D), increased significantly in response to heat stress in both jejunum and ileum, while this effect was more obvious in chicken ileum. Only in the jejunum of heat-exposed animals, a preventive effect on these increased cytokine mRNA levels was observed after feeding the GOS diet (Fig 5A and 5C).

### The decline in IL-8 plasma levels after heat stress exposure can be prevented by dietary GOS

In chickens exposed to heat stress, the IL-8 plasma levels were markedly decreased in comparison with control animals (Fig 5F), while no significant decrease in IL-6 secretion was detected (p = 0.31) (Fig 5E). The plasma levels of heat-exposed chickens fed a GOS diet, did contain more IL-8 compared to heat-exposed animals fed a control diet (Fig 5F).

### Heat stress increases the HIF-1α mRNA levels in ileum

**Fig 6:**
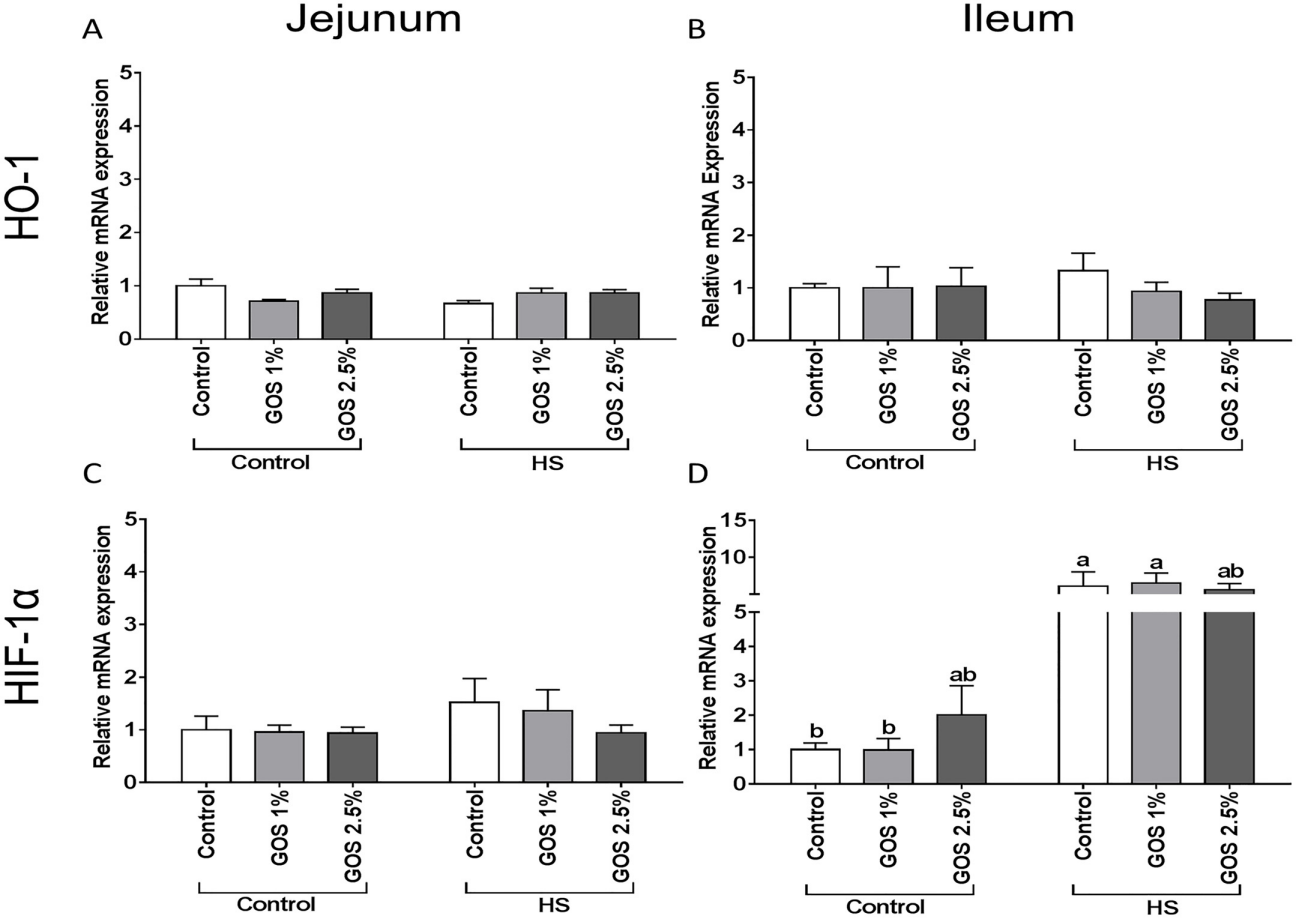
The effect of heat stress exposure on the mRNA expression of HO-1 and HIF-1α in jejunum and ileum of chickens fed a control or GOS diet. Chickens fed a control or GOS (1 or 2.5%) diet for 6 days before being exposed to either control or heat stress conditions for 5 days. The mRNA expression of HO-1 (A, B) and HIF-1α (C, D) in jejunum (A, C) and ileum (B, D) measured by qRT-PCR. Results are expressed as relative mRNA expression (fold of control, normalized to β-actin) as mean ± SEM, n = 6–10 animals/experimental group. Different lower-case letters denote significant differences among groups.

No remarkable changes were observed in the mRNA expression of the oxidative stress markers, haem oxygenase-1 (HO-1) and hypoxia inducible factor, subunit alpha (HIF-1α) in chicken jejunum after heat stress exposure (Fig 6A and 6C). In ileum, HIF-1α mRNA levels increased significantly after 5 days heat stress, while no effect of HO-1 mRNA expression was detected (Fig 6B and 6D). This increase in HIF-1α mRNA expression in ileum was not attenuated by GOS (Fig 6D).

## Discussion

High ambient temperatures can potentially induce patho-physiological alterations in humans and animals [29]. In particular, the gastrointestinal tract is considered as one of the main target organs affected by heat stress. The multifaceted response to heat stress is initiated by HSFs, regulating the expression of HSPs [30,31]. In this study, the gene expression of HSF3, an avian-specific member of the HSF family, was significantly increased after heat exposure in chicken jejunum and ileum, while the HSF1 mRNA levels were up-regulated in the ileum. Previous investigations in chicken embryo fibroblasts described that in addition to differences in DNA binding capacity, HSF1 and HSF3, also have different threshold temperatures for activation upon heat exposure [32]. More recently, Xie et al., [30] described a tissue specific pattern of heat-induced HSF1 and HSF3 expression, which may be related to the level of oxidative damage. In consideration of these findings, we analyzed the mRNA expression levels of the oxidative stress markers, HO-1 and HIF-1α, which are known to play a crucial role in response to heat-induced oxidative stress and heat acclimation [33–35] and are characterized in chicken intestines [36]. A significant increase in HIF-1α mRNA levels was observed only in the ileum of heat-exposed chickens compared to control chickens.

Up-regulated and activated HSFs target the major heat inducible proteins such as HSP70 and HSP90 [37], which occupy a central role in the regulation of protein homeostasis during physiological and pathological conditions [38], and are currently considered as general markers of tissue injury [39,40]. The chickens subjected to heat stress showed a significant up-regulation of HSP70 and HSP90 in mRNA levels in both jejunum and ileum, while the corresponding protein expression was only increased significantly for HSP70, in jejunum as well as in the ileum. HSP70 plays a major role in the adaptive response to heat stress in broilers by improving the antioxidant capacity, inhibiting lipid peroxidation and increasing the activity of digestive enzyme activity [41,42]. In contrast to the results in jejunum and ileum, no heat stress-related alterations in HSP70 and HSP90 mRNA expression were observed in duodenum and colon, indicating again differences in the susceptibility of the individual parts of the intestines. These findings are in line with a study Zhang et al., [43] in which differences in the mRNA expression of HSF3 and HSP70 between two chicken breeds and tissue-specific differences during heat treatment are described.

Another important adaptive response of the body to heat stress is increasing the peripheral blood flow, in turn resulting in a reduced blood supply in the intestines, and an hypoxia induced oxidative stress response. Moreover, the gut epithelial barrier becomes ischemic, leading to a dysfunctions of AJ and TJ and loss of barrier integrity. [44]. In this study, only in chicken jejunum, a significant increase in E-cadherin mRNA levels was detected after heat exposure, which could be regarded as a compensatory response related to the observed decrease in cadherins protein expression. Lang et al., [45] demonstrated that the E-cadherin gene and protein expression has been decreased in heat-exposed human lung adenocarcinoma cells. The link between E-cadherin and HSP expression was recently confirmed by Chen et al., observing a down-regulation of E-cadherin protein levels in colorectal cancer cells directly exposed to rHSP90α [46]. The *in vivo* data with respect to E-cadherin is in agreement with our recently published *in vitro* results with Caco-2 cells exposed to heat stress [47]. In addition to E-cadherin, heat exposure induced alterations in the level of mRNA expression of the TJ proteins claudin-5 and ZO-1, which was significantly increased in jejunum and ileum, and claudin-1 was upregulated in the ileum. The occludin mRNA levels remained unaffected after heat stress, which is in line with our previous results showing that in a Caco-2 cell model, the expression of TJ proteins remained unaffected by heat stress [47]. The absence of the microbiota in this Caco-2 *in vitro* model, that might play a role in the regulation of tight junction permeability during heat stress [48], could be a possible explanation for the discrepancies between the *in vitro* and *in vivo* findings. A study in pigs investigated the effect of heat stress on the protein expression of TJs in the ileum and showed an increase in claudin-3 and occludin protein expression in the heat-exposed animals, while no differences in claudin-1 expression were observed [29].

Disruption of these junctional proteins may lead to increased permeability of the intestinal barrier to luminal antigens, which will initiate inflammatory signaling via TLRs [12,13]. Although in this study, no significant changes were observed in TLR-2 mRNA expression along the intestine after heat exposure, the TLR-4 mRNA expression was significantly up-regulated in both chicken jejunum and ileum. This TLR-4 up-regulation could be induced by the invasion of Gram-negative bacteria due to the disrupted intestinal barrier [49] or by a direct effect of the heat exposure, since TLR4 has been described as a stress-related biosensor in initial injury responses {reviewed in [14]}. Furthermore, TLR-4 activation can contribute to the intestinal barrier breakdown, since it has been demonstrated that TLR-4 knockout mice were protected from burn-induced intestinal hyperpermeability [13].

The heat stress-induced changes in HSPs, HIF-1α, junctional proteins and TLR-4 expression levels, were accompanied with an inflammatory reaction in the intestine, since chickens exposed to heat stress, showed significantly higher mRNA expression level of IL-6 and IL-8 in both jejunum and ileum, whereas the production of these cytokines in jejunum and ileum remained unchanged (S3 Fig). Independent of the local effects on IL-6 and IL-8 mRNA levels under heat stress conditions, probably caused by the intestinal barrier disruption, penetration of pathogens and exaggerating the extent of TLR-4 signaling, a decrease in IL-8 plasma levels was observed after heat stress exposure. This could be related to the anti-inflammatory effects of HSPs within a heat shock response [20,50], as HSPs are activators of anti-inflammatory regulatory T cells [51] and HSP induction most probably blocks the NF-kB activation by stabilizing IκBα [21]. In addition, it is known that HSFs can also display anti-inflammatory properties [52].

Overal, the increased mRNA expression levels of the HSFs, HSPs, TJ proteins, TLR-4, IL-6 and IL-8 were more pronounced in ileum of heat-exposed chickens compared to the jejunum, suggesting the higher susceptiblity of the ileum to heat-induced oxidative stress as the mRNA expression of the oxidative stress marker, HIF-1α, was only up-regulated in ileum. Although heat stress-induced injury has been previously described in the chicken small intestine [7,53], no attention was paid to the differences in susceptibility of individual intestinal segments. However, Santos et al., [54] showed the influence of heat stress on morphological parameters of the duodenal, jejunal and ileal mucosa.

A possible explanation for the more severe heat-induced damage observed in ileum could be related to the difference in the microbiota composition between the ileum and jejunum [55,56] and the related anti-inflammatory effects of the microbial HSPs and their related peptides [51,57].

In this study we hypothesized that dietary GOS, supporting the gut homeostasis, may exert protective effects against the heat stress-induced multifaceted response in the chicken intestine. In the chicken jejunum, all heat-stress induced changes (HSFs, HSPs, E-cadherin, TJ proteins, TLR-4, IL-6 and IL-8) were prevented by the GOS supplemented diet, resulting in a less severe heat stress response. However, GOS supplementation failed to modulate the heat-induced changes in the chicken ileum.

Since GOS has been associated with stimulating the beneficial microbial population [26,58], the protective effect of GOS after heat stress could be related to changes in microbial HSPs induced by GOS. However, GOS could also prevent the heat-induced up-regulation of HSP70 and HSP90 in the Caco-2 monolayer lacking the microbial environment [47]. The latter findings suggest that GOS has also direct effects on the expression of TJ proteins [19]. Microbiota-independent mechanisms by direct interaction on immune and epithelial cells have been confirmed recently [59–61]. Moreover, the observed effects of GOS might be similar to the macromolecule-stabilizing character of some sugars like trehalose-based oligosaccharides, that act as antioxidants [62,63]. From different *in vivo* studies it is known that supplementation of the diet with non-digestible oligosaccharides like GOS and mannan-oligosaccharides (MOS) improved the intestinal integrity [28,64] and exert direct immuno-modulatory effects (reviewed for GOS/ fructo-oligosaccharides by Jeurink et al., 2013 [65]). In chickens, an improvement of the intestinal mucosal architecture as observed in a study with dietary supplementation of MOS and probiotics mixtures [66] and fructo-oligosaccharides, inulin and MOS are also described to reduce the susceptibility of chicken intestine to colonization by pathogenic bacteria, like *Salmonella* spp. [67,68]. Further investigations should aim to understand the possible direct and indirect mechanisms underlying the integrity- and immune-regulating effects of GOS associated to intestinal injury.

## Conclusions

Our results provide for the first time evidence for a difference in susceptibility of individual intestinal segments to heat stress as demonstrated by the assessment of different biomarkers including HSPs, HSF, AJ and TJ, cytokines and oxidative stress markers. Alterations in the level of expression of these biomarkers was more pronounced in ileum of heat-exposed chickens compared to the jejunum. Dietary application of GOS, an oligosaccharide that acts as a prebiotic, but also exerts direct, microbiota-independent effects and stabilizes intestinal integrity, could not mitigate the alterations in the ileum, but succesfully prevented all heat-stress induced changes (HSFs, HSPs, E-cadherin, TJ proteins, TLR-4, IL-6 and IL-8) in the jejunum.

## Supporting Information

S1 Fig The effect of heat stress exposure on the mRNA expression of HSPs in duodenum and colon of chickens fed a control or GOS diet. Chickens fed a control or GOS 2.5% diet for 6 days before being exposed to control or heat stress conditions for 5 days. The mRNA expression of HSP70 and HSP90 was quantified in duodenum (A, C) and colon (B, D) by qRT-PCR. Results are expressed as relative mRNA expression (fold of control, normalized to β-actin) as mean ± SEM, n = 10 animals/experiment group. Different lower-case letters denote significant differences among groups.
(TIF)

S2 Fig The effect of heat stress exposure on the mRNA expression of occludin in jejunum and ileum of chickens fed a control or GOS diet. Chickens fed a control or GOS 2.5% diet for 6 days before being exposed to control or heat stress conditions for 5 days. The mRNA expression of occludin was quantified in jejunum (A) and ileum (B) by qRT-PCR. Results are expressed as relative mRNA expression (fold of control, normalized to β-actin) as mean ± SEM, n = 6–10 animals/experiment group. Different lower-case letters denote significant differences among groups.
(TIF)

S3 Fig The effect of heat stress exposure on IL-6 and IL-8 production in jejunum and ileum of chickens fed a control or GOS diet. Chickens fed a control or GOS 2.5% diet for 6 days before being exposed to control or heat stress conditions for 5 days. ELISA was performed for quantification of IL-6 and IL-8 production in jejunum (A, C) and ileum (B, D) homogenates. Results are expressed in pg/ml as mean ± SEM, n = 5 animals/experimental group.
(TIF)

S1 Table Feed composition standard broiler diet. (PDF)

S2 Table Primer sequences used for qRT-PCR. (PDF)
