# Supplementary figures and images for: Differences in Susceptibility to Heat Stress along the Chicken Intestine and the Protective Effects of Galacto-Oligosaccharides
Source: PLoS One. 2015 Sep 24;10(9):e0138975. doi: 10.1371/journal.pone.0138975 (PMC4581695; doi:10.1371/journal.pone.0138975)

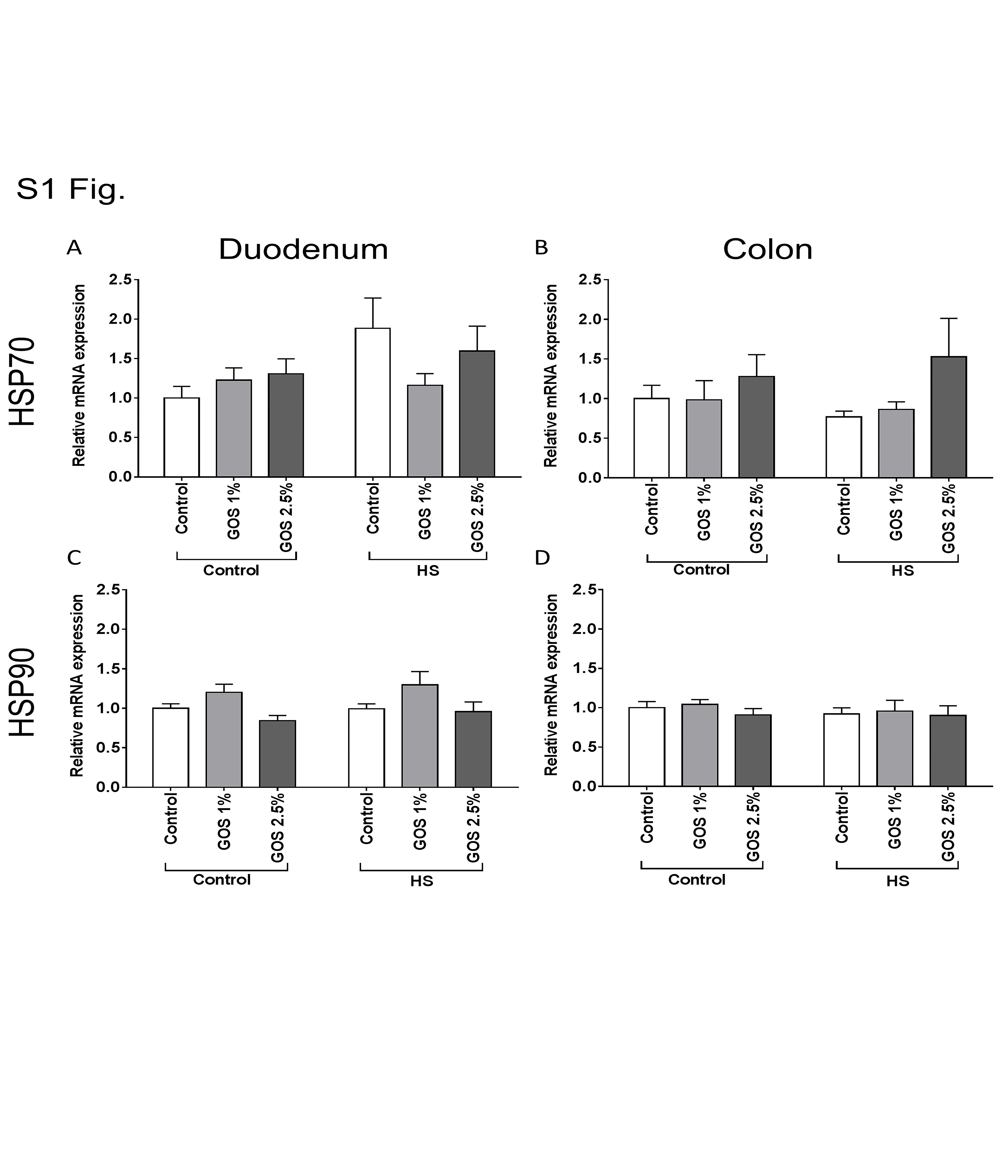

Supplement: S1 Fig — Chickens fed a control or GOS 2.5% diet for 6 days before being exposed to control or heat stress conditions for 5 days. The mRNA expression of HSP70 and HSP90 was quantified in duodenum (A, C) and colon (B, D) by qRT-PCR. Results are expressed as relative mRNA expression (fold of control, normalized to β-actin) as mean ± SEM, n = 10 animals/experiment group. Different lower-case letters denote significant differences among groups. (TIF) [file pone.0138975.s001.tif]

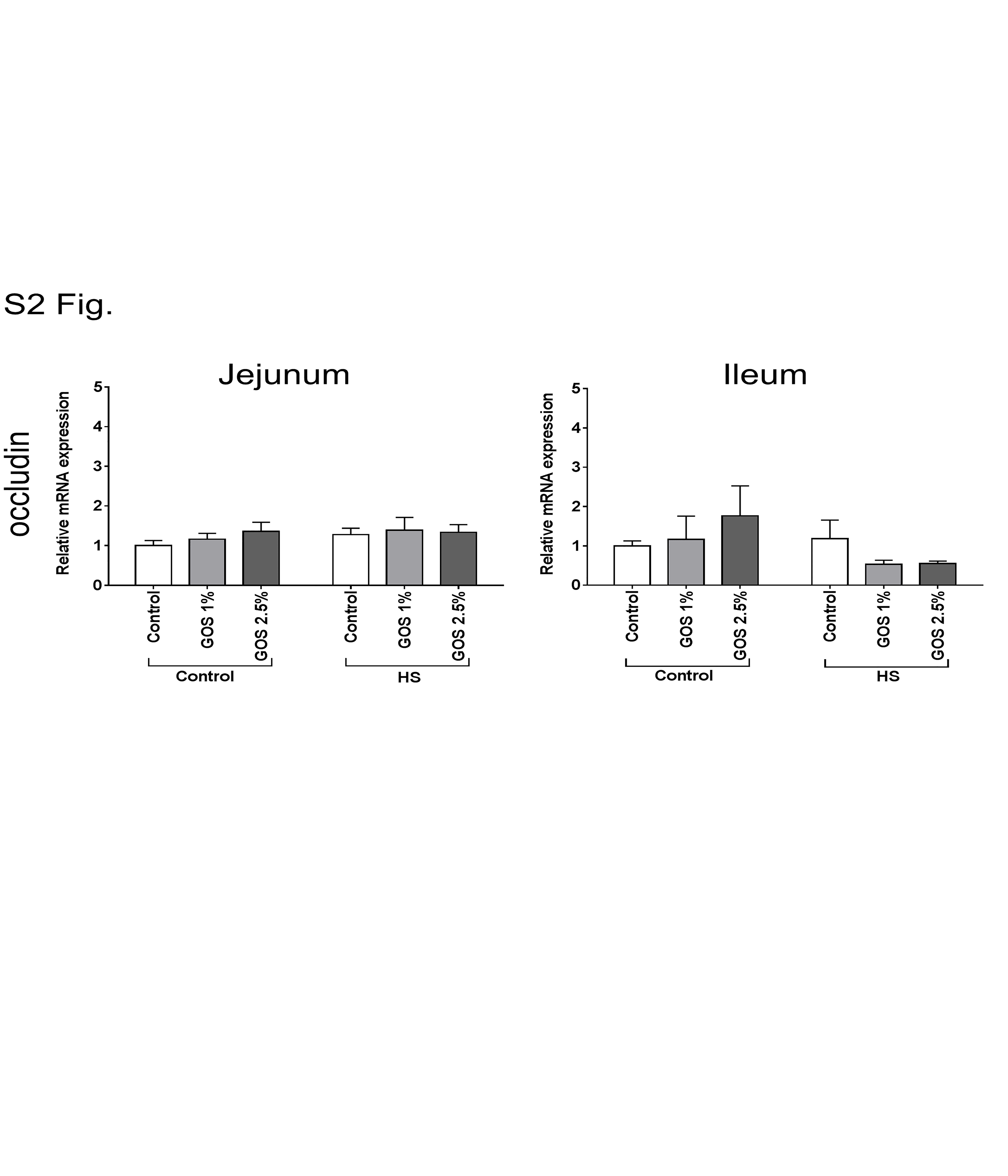

Supplement: S2 Fig — Chickens fed a control or GOS 2.5% diet for 6 days before being exposed to control or heat stress conditions for 5 days. The mRNA expression of occludin was quantified in jejunum (A) and ileum (B) by qRT-PCR. Results are expressed as relative mRNA expression (fold of control, normalized to β-actin) as mean ± SEM, n = 6–10 animals/experiment group. Different lower-case letters denote significant differences among groups. (TIF) [file pone.0138975.s002.tif]

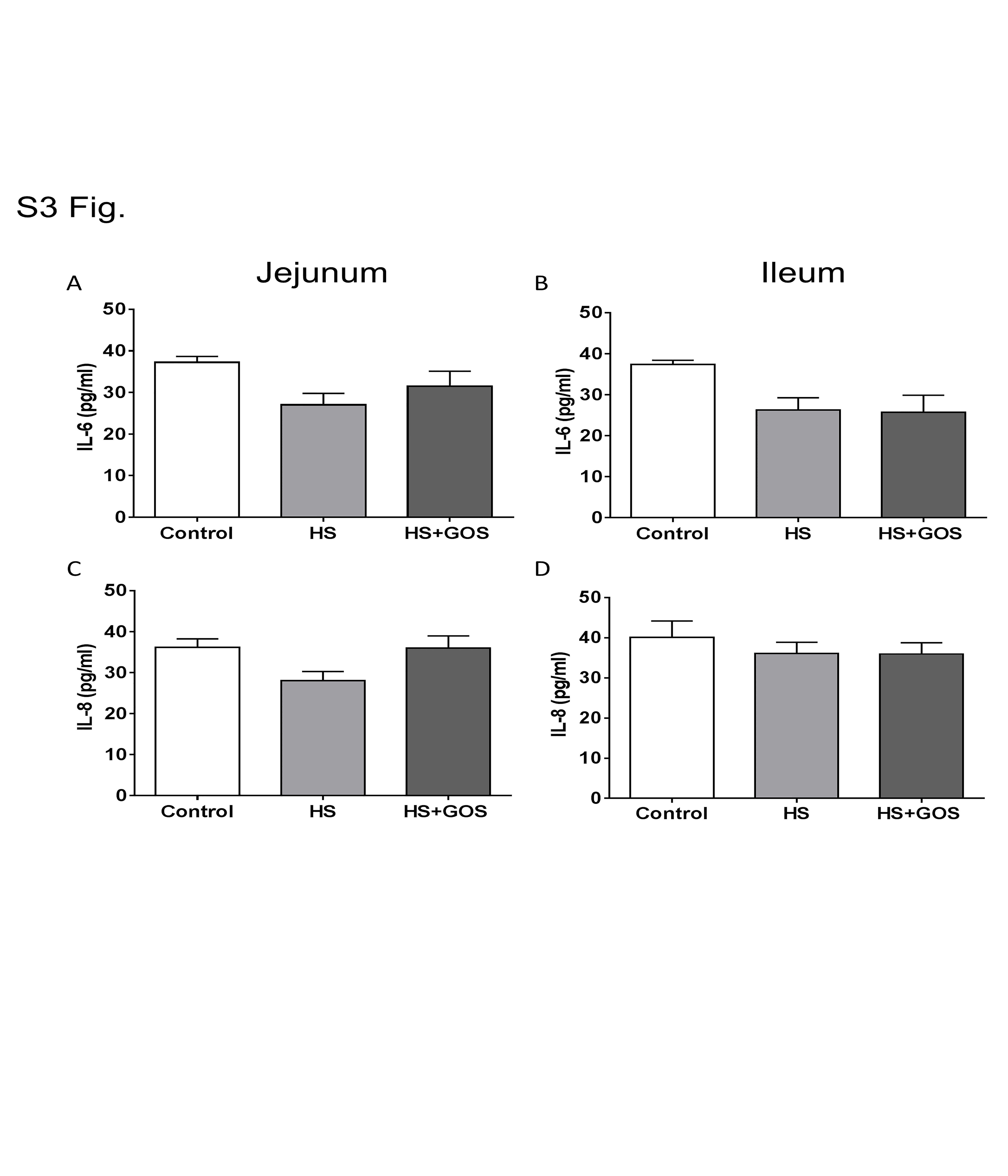

Supplement: S3 Fig — Chickens fed a control or GOS 2.5% diet for 6 days before being exposed to control or heat stress conditions for 5 days. ELISA was performed for quantification of IL-6 and IL-8 production in jejunum (A, C) and ileum (B, D) homogenates. Results are expressed in pg/ml as mean ± SEM, n = 5 animals/experimental group. (TIF) [file pone.0138975.s003.tif]
