# Supplementary material for: Differences in Susceptibility to Heat Stress along the Chicken Intestine and the Protective Effects of Galacto-Oligosaccharides
Source: PLoS One. 2015 Sep 24;10(9):e0138975. doi: 10.1371/journal.pone.0138975 (PMC4581695; doi:10.1371/journal.pone.0138975)
Supplement: S1 Table — (PDF) [file pone.0138975.s004.pdf]

S1 Table.

Ingredients	Value (g/kg)	Nutrients	Value (g/kg)	Amino Acids	Total (g/kg)
Maize	322,20	Dry matter	879,60	Isoleucine	8,30
Wheat + xylanase	250,00	Crude ash	50,10	Leucine	15,93
Soya	225,00	Crude protein	204,20	Lysine	11,75
Rapeseed meal	30,00	Crude fat	70,80	Methionine	5,19
Peas (dry)	50,00	Crude fiber	30,50	Cystine	3,44
Soy bean (heated)	50,00	Carbohydrates	525,30	Phenylalanine	9,67
Lard	20,00	Organic matter	146,00	Tyrosine	6,95
Soy bean oil	20,00	Starch	389,20	Threonine	7,96
Premix (maize)	5,00	Sugars	40,40	Tryptophan	2,33
Chalk	13,00	Neutral detergent fibre	99,30	Valine	9,34
Limestone	0,00	Acid detergent fibre	39,70	Arginine	13,14
Monocalcium phosphate	6,00	Calcium	7,90	Histidine	5,29
Salt (NaCl)	2,20	Total phosphorus	4,90	Alanine	9,33
NaHCO3	2,00	Magnesium	1,60	Aspartic acid	19,42
Phytase	0,10	Potassium	9,00	Glutamic acid	38,27
L-lysine HCL	1,70	Sodium	1,48	Glycine	8,39
DL-methionine	2,20	Chlorine	2,00	Proline	12,33
L-threonine	0,60	Base-excess (mEq/kg)	235,50	Serine	9,85
		Net energy (MJ/kg)	10,40		
		Linoleic acid (MJ/kg)	26,80		
		Fe (mg/kg)	136,00		
		Mn (mg/kg)	21,00		
		Zn (mg/kg)	30,00		
		Cu (mg/kg)	6,00		
