# Supplementary material for: Differences in Susceptibility to Heat Stress along the Chicken Intestine and the Protective Effects of Galacto-Oligosaccharides
Source: PLoS One. 2015 Sep 24;10(9):e0138975. doi: 10.1371/journal.pone.0138975 (PMC4581695; doi:10.1371/journal.pone.0138975)
Supplement: S2 Table — (PDF) [file pone.0138975.s005.pdf]

S2 Table.

	Primer sequence (5’-3’)			
**Gene**	**Forward**	**Reverse**	**AT**	**References**
**ACTB**	ATGTGGATCAGCAAGCAGGAGTA	TTTATGCGCATTTATGGGTTTTGT	61	NM_205518.1
**Claudin-1**	CTGATTGCTTCCAACCAG	CAGGTCAAACAGAGGTACAGG	58	NM_001013611
**Claudin-5**	CATCACTTCTCCTTCGTCAGC	GCACAAAGATCTCCCAGGTC	58	NM_204201
**E-cadherin**	GACAGGGACATGAGGCAGAA	GCCGTGACAATGCCATTCTC	64.3	NM_001039258.2
**HIF-1α**	ACCATTACCATACTTCAGCAG	CTTCACATCATCCAGACGTTC	65	NM_204297
**HO-1**	CTTCGCACAAGGAGTGTTAAC	CATCCTGCTTGTCCTCTCAC	63	NM_205344
**HSF1**	CAGGGAAGCAGTTGGTTCACTACACG	CCTTGGGTTTGGGTTGCTCAGTC	65	L06098.1
**HSF3**	TCCACCTCTCCTCTCGGAAG	CAACAGGACTGAGGAGCAGG	57	L06126.1
**HSP70**	TCTCATCAAGCGTAACACCAC	TCTCACCTTCATACACCTGGAC	55	JX827254.1
**HSP90**	ATGCCGGAAGCTGTGCAAACACAGGACCAA	GGAATCAGGTTAATTTTCAGGTCTTTTCCA	63.1	NM_001109785.1
**IL-6**	GCTCGCCGGCTTCGA	GGTAGGTCTGAAAGGCGAACAG	58.7	HM179640.1
**IL-8**	CACGTTCAGCGATTGAACTC	GACTTCCACATTCTTGCAGTG	61.2	NM_205018.1
**occludin**	ACGGCAGCACCTACCTCAA	GGGCGAAGAAGCAGATGAG	61.2	D21837.1
**TLR-2**	CCTGCAACGGTCACTTCAG	GTCTCAGGGCTTGTTCTTCAG	59	NM_204278
**TLR-4**	CTGACCTACCCATCGGACAC	GCCTGAGAGAGGTCAGGTTG	59	NM_001030693
**ZO-1**	CTTCAGGTGTTTCTCTTCCTCCTC	CTGTGGTTTCATGGCTGGATC	59	XM_413773
